# S79. CAR strategies in solid tumours

**DOI:** 10.1186/2051-1426-2-S2-I17

**Published:** 2014-03-12

**Authors:** C Rossig

**Affiliations:** 1 Klinik und Poliklinik fur Kinderheilkund, Dept. of Pediatric Hematology and Oncology, Münster, Germany

## 

The development of immune-based strategies for cancer for solid cancers is challenged by the scarcity of T cells with high receptor avidity for tumor-specific antigens within the patient´s lymphocyte repertoire, and by the failure of tumor cells to present antigen to T cells. Both obstacles can be bypassed by genetic modification of T cells with recombinant chimeric receptors (CARs) which redirect T cells towards a tumor surface antigen independent of antigen presentation. CAR reengineered T cells efficiently interact with tumor cells in vitro and have significant in vivo activity against tumor xenografts. Recently, first clinical trials have shown evidence for a potent antitumor activity of CD19-specific CAR T cells in leukemia. Current efforts focus on improving in vivo survival, functional persistence and potency of adoptively transferred anti-tumor T cells. The design of more effective strategies against both solid tumors and leukemias further depends on enhanced knowledge of specific mechanisms of immune escape. Moreover, rational combinations of targeted therapies with immunotherapies and optimal integration of cellular therapies into current treatment regimens may allow higher rates of durable responses.

